# Kinetics and Specificity of HEK293T Extracellular Vesicle Uptake using Imaging Flow Cytometry

**DOI:** 10.1186/s11671-020-03399-6

**Published:** 2020-08-24

**Authors:** Brian J. Jurgielewicz, Yao Yao, Steven L. Stice

**Affiliations:** 1 Regenerative Bioscience Center, University of Georgia, Athens, GA 30602 USA; 2 Department of Animal and Dairy Science, University of Georgia, Athens, GA 30602 USA; 3 ArunA Bio, Athens, GA 30602 USA

**Keywords:** Extracellular vesicles, Selective uptake, HEK293T, Imaging flow cytometry

## Abstract

Extracellular vesicles (EVs) are nanosized lipid bilayer-bound vesicles that are naturally secreted from most cell types as a communication mechanism to deliver proteins, lipids, and genetic material. Despite the therapeutic potential of EVs, there is limited information on EV uptake kinetics and specificity. Here, we optimized an imaging flow cytometry (IFC)-based platform to quantitatively assess dose, time, and recipient cell specificity effects on human embryonic kidney cell (HEK293T) EV internalization in a high-throughput manner. We found that HEK293T EV uptake is an active process that is dose and time dependent. Further, the selectivity of EV uptake was quantified *in vitro*, and we found that HEK293T EVs were internalized at higher quantities by cells of the same origin. Lastly, neural stem cells internalized significantly more HEK293T EVs relative to mature neurons, suggesting that stem cells or progenitors, which are more metabolically active than terminally differentiated cells, may have higher rates of active EV internalization. The characterization of EV uptake, notably specificity, dose and time dependence, and kinetic assays will help inform and develop targeted and efficient EV-based therapeutics.

## Introduction

Extracellular vesicle research is a burgeoning field due to the therapeutic and diagnostic utility of natural and engineered extracellular vesicles (EVs). EVs range from 50 to 1000 nm in diameter, are produced from all cell types, and are enriched with transmembrane proteins, including CD63, CD81, and CD9; lipids; proteins; and DNA, RNA, mRNA, and microRNA [1–5]. EV content, notably active mRNA and miRNA, has been implicated in modulation of recipient cells via de novo translation and post translational regulation of target cells [4, 6]. Understanding and then modifying kinetic EV uptake and internalization will eventually lead to optimized delivery of EV contents to target cells with high-enough concentrations to have a therapeutic benefit.

Once thought to be “the garbage of the cells”, EVs have been harnessed as an alternative to cell therapies due to many advantages including their biocompatibility, low immunogenicity and toxicity, ability for repeated dosing, various routes of administration, and potential to deliver drugs and genetic therapies [3]. Our group has previously reported positive effects of neural stem cell-derived EVs in stroke and traumatic brain injury. In both murine and porcine stroke models, EVs improved tissue and functional recovery post stroke [3, 7, 8]. We have also shown EVs to be neuroprotective with functional benefits in a rodent traumatic brain injury model [9]. Despite these observed effects and future potential of EVs, there is little understanding of EV uptake specificity and kinetics, which may hinder translation of EV therapeutics into the clinic.

EVs have also been engineered as transference vectors and loaded with therapeutic agents including gene therapies and chemical compounds as an alternative to nanoparticle therapeutics and delivery vectors [4, 10–12]. HEK293T cells have been widely used as EV producer cells due to their inherent rapid proliferation, high EV yield, and ease of genetic manipulation [13–17]. HEK293T EVs delivered gene therapies including miRNA therapeutics for breast cancer [12] and have been used to deliver chemotherapeutics and therapeutic protein constructs in a schwannoma model [18]. Similar to synthetic nanoparticle studies assessing cytotoxicity in vitro, MTT toxicity assays displayed low toxicity of unloaded HEK293T EVs and subsequent high cytotoxicity when loaded with chemotherapeutics [10, 18–21]. Due to this abundant utilization of HEK293T EVs, we analyzed their kinetics and specificity in this study.

Selective or specific uptake refers to an EV’s natural ability to target specific cell types. There is abundant evidence on the mechanisms of EV internalization with little consensus on uptake specificity [22]. Often EVs exhibit selective uptake by similar recipient cells as their parent cells, epithelial cells internalize more epithelial-derived EVs than other recipient cells [23, 24], and mesenchymal stem cells (MSC) internalize a significantly greater amount of MSC-derived EVs compared with other cell lines in vitro [24]. However, other studies found that EVs are internalized by all cell types and display a non-selective biodistribution when administered in vivo [22, 25]. Despite the immense therapeutic potential and interest of EVs, there is a deficiency in the understanding of EV uptake specificity. By better understanding EV uptake specificity, we can appropriately choose EV producer cells that are selectively internalized by recipient cells of interest and thus improve the therapeutic applicability of EVs.

A potential reason for conflicting EV uptake results is the lack of standardization in measurement platforms, including analyses of dose and time effects. Recently, an International Society for Extracellular Vesicles (ISEV) group of experts released a position paper emphasizing the need for analysis of dose and time, amongst other confounding factors on EV uptake [26]. The group stated that “one dose does not fit all” and that dose may affect EV uptake or selectivity [26]. Elevating doses of HEK293T EVs shifts the biodistribution pattern in vivo [27]. Uptake profiles of serum-derived EVs were significantly altered by dose [28]. Additionally, co-incubation times of EVs with recipient cells ranging from 15 min to 48 h [24, 29–33] may alter uptake measurements. If adopted by EV researchers and industry, a quantifiable and reliable process to determine standard dose and time curves to help identify the minimum effective dose may lead to more robust and useful studies.

Previously, researchers have used standard flow cytometry along with various forms of low-throughput microscopy including confocal microscopy to analyze EV uptake [32–34]. However, these technologies have several limitations. Confocal microscopy can be time consuming and subjective. Traditional flow cytometers have been designed to measure biological particles in the cellular range, cannot differentiate EV swarm or coincidence, and have increased noise due to triggering [35–38]. As mentioned by the ISEV group, there is growing awareness of the physical limitations of traditional flow cytometry and highlight the demand for specialized flow cytometry with detection limits in the 100-nm range [26, 38]. Imaging flow cytometry (IFC) combines the high-throughput quantitative nature of flow cytometry along with fluorescence imaging technology which can resolve inherently small fluorescent particles, down to 100 nm in diameter [38]. IFC capabilities lead to low noise/background, decreased swarming, and charged coupled devices for image clarity [37, 39]. These characteristics assist in developing a gating strategy for characterizing EVs and uptake with visual confirmation in a high-throughput manner as an accurate and quantifiable EV uptake platform [36, 37, 40].

In this study, CD63-eGFP–expressing HEK293T cells were utilized as the donor cell line for EV production due to their common usage in therapeutic development. The isolated fluorescent EVs were co-cultured with recipient cell lines including neural and endothelial cells. Uptake was quantified using IFC, resulting in a standardized platform to measure the important kinetic EV uptake and internalization features for in vitro cell systems. Further, we provide data on a process to quantify uptake of fluorescent EV uptake in differing conditions and cultured cell lines to elucidate selective EV uptake.

## Materials and Methods

### Cell Culture

Human embryonic kidney cells (HEK293T) were purchased from ATCC and cultured in DMEM containing 10% fetal bovine serum, 100 U/mL penicillin, and 100 μg/mL streptomycin. Human neural stem cells (hNSC), SH-SY5Y neural cells, C3A liver epithelial cells, human umbilical vein endothelial cells (HUVEC), and neurons were all cultured under standard conditions at 37 °C, 5% CO_2_ prior to extracellular vesicle uptake assays.

### EV Labeling and Isolation

CD63-eGFP plasmid DNA was obtained from Addgene (#62964). CD63-pEGFP C2 was a gift from Paul Luzio (Addgene plasmid #62964). HEK293T cells were cultured to 70% confluency in 10-cm dishes, and 10 μg plasmid DNA was transfected using Lipofectamine 2000 according to the manufacturer’s instructions. Twenty-four hours post transfection, media were changed to standard HEK293T media devoid of fetal bovine serum and collected for 3 consecutive days. As previously described [3], HEK293T media were filtered through a 0.22-μm filter and enriched by ultrafiltration using a 100-kDa regenerated cellulose Amicon centrifugal filter units and washed twice with PBS++. EVs were concentrated to 1 mL, and concentration and size distributions were measured on Nanosight NS300 by the manufacturer’s protocol (Malvern, UK). EVs were isolated from different HEK293T culture vessels, each vessel considered separate biological replicates, with three technical replicates within each biological replicate (minimum of nine samples total for each condition).

### Uptake Assays

Recipient cell lines were seeded at 60% confluency in a 6-well plate for 24 h under standard culture conditions at 37 °C. Standard media were changed to fetal bovine serum-free (FBS−) media prior to extracellular vesicle co-culture. Green fluorescent protein (GFP)-tagged EVs were administered to cells at varying doses and time points. After co-culture, cells were resuspended in 5% trypsin and concentrated to around one million cells per 50 μL for flow cytometry. Thirty-seven degrees Celsius is the standard for EV uptake experiments in our assays as it has been the standard used for both cell culture and in vitro EV uptake platforms [31, 41–44].

### Inhibitory Assays

#### Cold Assay

EVs were co-cultured with recipient cells at 4 °C to effectively “pause” cell culture growth and inhibit active processes [45]. Four degrees Celsius inhibits all active forms of EV uptake [31, 41–44].

#### Fixed Assay

Recipient cells were fixed in 4% paraformaldehyde for 30 min on ice and washed with PBS immediately before co-culture with EVs to inhibit all active forms of EV uptake.

### ImageStreamX Acquisition

Acquisition was performed on the ImageStreamX Mark II Imaging Flow Cytometer (Luminex Corporation, Seattle, Washington) using the INSPIRE software. A minimum of 5000–10,000 cell events were acquired. Each biological sample was replicated in three technical replicate wells and individually acquired on the ISx. Bright field images were collected on channel one and side scatter (785 nm) on channel six. Green fluorescent protein (GFP) was excited by 488 nm argon laser at 200 mW, and fluorescence was collected on channel two (480-560 nm). A magnification of 60× was used on every sample along with a low acquisition rate for high sensitivity.

### IDEAS Analysis

Data and image analyses were conducted using the IDEAS software (Luminex). The gating strategy is the following:

1. Focus gate was determined to eliminate cells that were not in the field of focus using the Gradient RMS value.
2. The focused cells were gated to eliminate doublets and debris using area bright field vs. aspect ratio bright field. Gated data were used to create histograms and generate statistic references measuring fluorescence intensity (sum of all pixels in an image), maximum pixel intensity (intensity of the brightest pixels in an image), along with spot count values via internal algorithms for every sample. Spot count features were generated using the applicable IDEAS Wizards. Spot count, mean intensity, and maximum pixel ratio are calculated by the formula (Output value with EVs/Output value without EVs).

### Statistics

All quantitative data were analyzed via GraphPad Prism 8.1.2 (San Diego, California) and done in triplicates. The data are presented as mean ± standard error of the mean (SEM). Statistical significance was determined using an unpaired *T* test or a one-way analysis of variance (ANOVA) with Tukey’s or Dunnett’s multiple comparison post hoc compared with controls when appropriate. *p* < 0.05 was considered significant.

## Results

### CD63-eGFP–Tagged HEK293T Extracellular Vesicle Properties

**Fig. 1:**
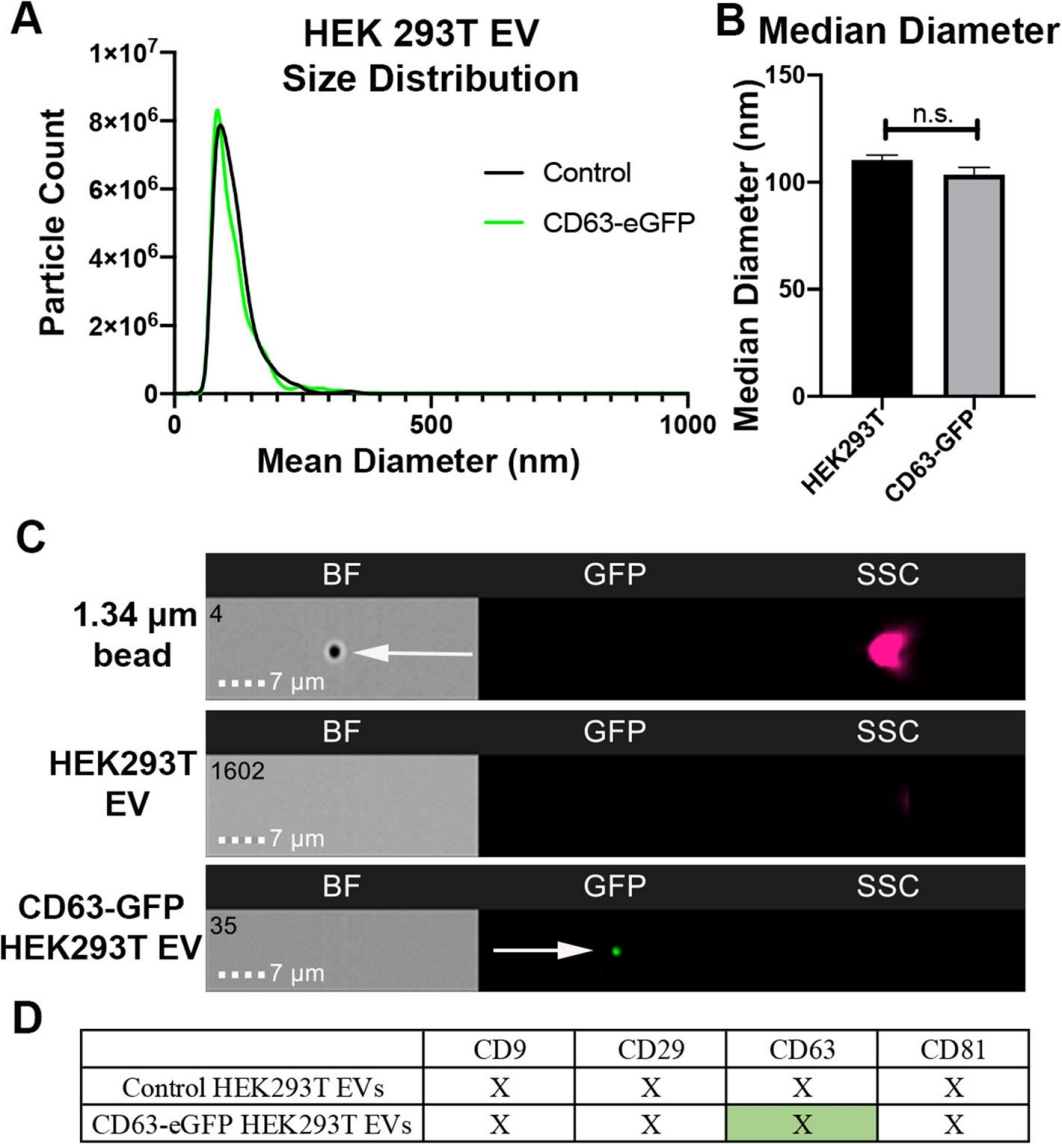
Characterization of HEK293T EVs tagged with CD63-eGFP. EVs were isolated from HEK293T (control) and HEK293T expressing CD63-eGFP cell culture media. **a** Representative EV size distribution recorded via nanotracking software. **b** Quantification of mean diameter distribution of transfected vs. non-transfected HEK293T EVs. **c** IFC images of negative control beads, HEK293T control EVs, and CD63-eGFP–tagged EVs. BF signifies bright field, GFP signifies green fluorescent protein (488 nm excitation laser), and SSC signifies side scatter. Positive eGFP in GFP channel signifies fluorescent HEK293T EVs. **d** Flow cytometry-based MACSPlex surface marker expression of non-transfected HEK293T EVs and HEK293T CD63-eGFP EVs. Both EV sources are positive for CD29, CD9, CD63, and CD81, as measured in relative fluorescence (denoted by X for positive). Bars represent mean ± SEM; *N* = 3; unpaired T test. n.s. signifies *p* > 0.05

To generate fluorescently labeled EVs for analyzing the kinetics and uptake of extracellular vesicles, HEK293T cells were transfected with a plasmid carrying CD63-eGFP fusion protein. CD63 is a tetraspanin protein commonly enriched in the membrane of exosomes making it an optimal target for EV fluorescent tagging [46, 47]. Spent media were collected from HEK293T cell culture, and EVs were isolated as previously reported [8]. We compared the size and distribution of EVs isolated from CD63-eGFP–transfected HEK293T cells to non-transfected HEK293T cells. Control and CD63-eGFP–transfected HEK293T EVs displayed an average median diameter of 110.28 nm and 103.616 nm, respectively, as measured by nanotracking software (Fig. 1a), which is consistent with the reported size of HEK293T EVs [13, 15, 27, 48]. No significant differences in median diameter (*p* = 0.1615) and distribution (*p* = 0.4225) of EVs isolated from non-transfected and CD63-eGFP–transfected HEK293T cells were observed. eGFP labeling did not alter size of HEK293T EVs (Fig. 1b).

IFC assay was conducted to determine if the CD63-eGFP was associated with EVs. As a fluorescent negative control, 1.34 μM beads in buffer solution (Fig. 1 c, top) lacked fluorescence when exposed to the 488-nm excitation wavelength, but were visible in bright field (BF) and side scatter (SSC). Untagged HEK293T EVs were negative in the BF, GFP, and SSC, suggesting a small size below the BF threshold and lack of fluorescence (Fig. 1d, middle). The absence in BF signifies an EV size smaller than 300 nm, which suggests minimal swarming of EVs. Lastly, CD63-eGFP–tagged EVs are negative in BF and positive in the GFP channel signifying positive fluorescence of the HEK293T EVs (Fig. 1c, bottom). The positive signal in the GFP channel may be indicative of a single EV or a group of fluorescent EVs. Collectively, these results show that the isolated HEK293T EVs have standard size and protein marker profiles consistent with previous reports of HEK293T exosomes, and eGFP labeling does not alter the size of HEK293T EVs [5, 27].

Using a commercially available flow cytometry-based method to measure common EV markers, we determined the overall EV tetraspanin profile [5]. Isolated HEK293T EVs from control and CD63-eGFP–expressing HEK293T cells were positive for standard EV markers including CD9, CD63, and CD81 as measured in relative fluorescence units (Fig. 1d). As previously reported, CD29 was also found on the surface of HEK293T EVs and CD63-eGFP–transfected HEK293T EVs [5]. These results indicate that the isolation and tagging methods for HEK293T EV result in EVs with common HEK293T exosome markers.

### Active Uptake of HEK293T EVs

**Fig. 2:**
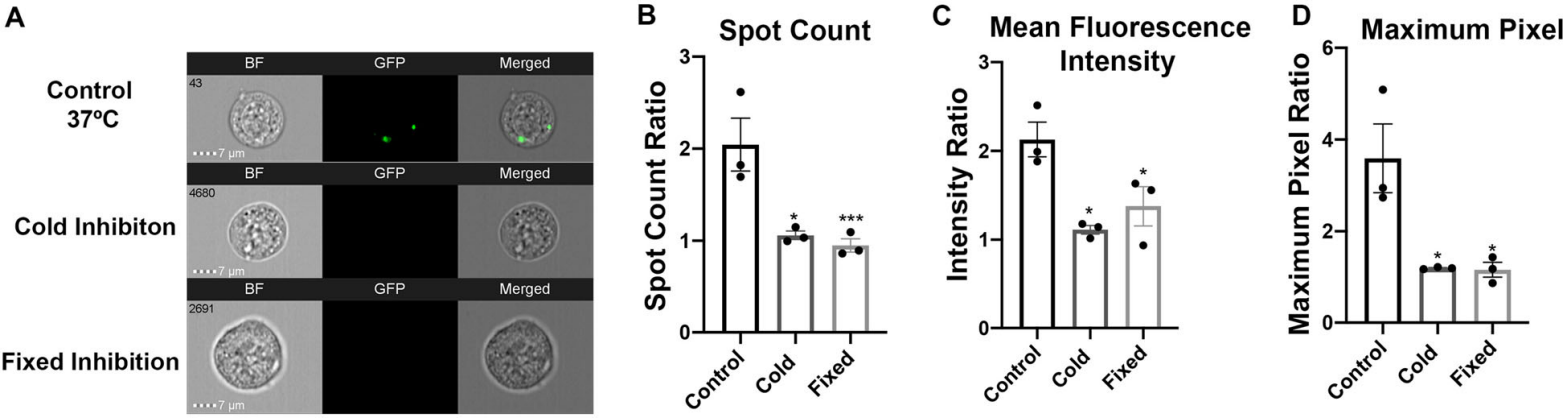
EV internalization inhibition assays. HEK293T cells were co-cultured with HEK293T EVs under various conditions. Control (37 °C) refers to co-culture in physiological 37 °C environment. Cold refers to co-culture in a 4 °C environment. Fixed inhibition refers to an assay where recipient cells were PFA fixed prior to co-culture. **a** Representative IFC images of recipient cells. Column 1, BF, signifies bright field. Column 2, GFP, signifies green fluorescent protein (488 nm excitation laser), and Column 3 signifies a merge of BF and GFP. Control shows positive GFP representing EV internalization. **b**–**d** Quantification of inhibition assays compared to controls via spot count, mean fluorescence intensity, and maximum pixel. Bars represent mean ± SEM; *N* = 3; one-way ANOVA followed with Tukey’s post hoc test compared with control. *p < 0.05; ***p < 0.01

Two inhibitory internalization assays were performed. HEK293T EVs were co-cultured with recipient cells at 4 °C (cold) or with recipient cells previously fixed with paraformaldehyde (fixed). The treatments decreased the presence of eGFP-labelled EVs in the recipient cells compared with recipient cells co-cultured with EVs under physiological conditions (Fig. 2a). Cold and fixed inhibitory assays reduced the spot count (cold: *p* = 0.0127, fixed: *p* = 0.0078), intensity (cold: *p* = 0.0105, fixed: *p* = 0.0374), and maximum pixel (cold: *p* = 0.0159, fixed: *p* = 0.0149) of fluorescence signals in recipient cells without treatments, indicating inhibition of EV uptake. These results infer that eGFP localization and increases in output parameters signify that HEK293T EVs are internalized for the following uptake assays.

### Dose-Dependent HEK293T EV Uptake

**Fig. 3:**
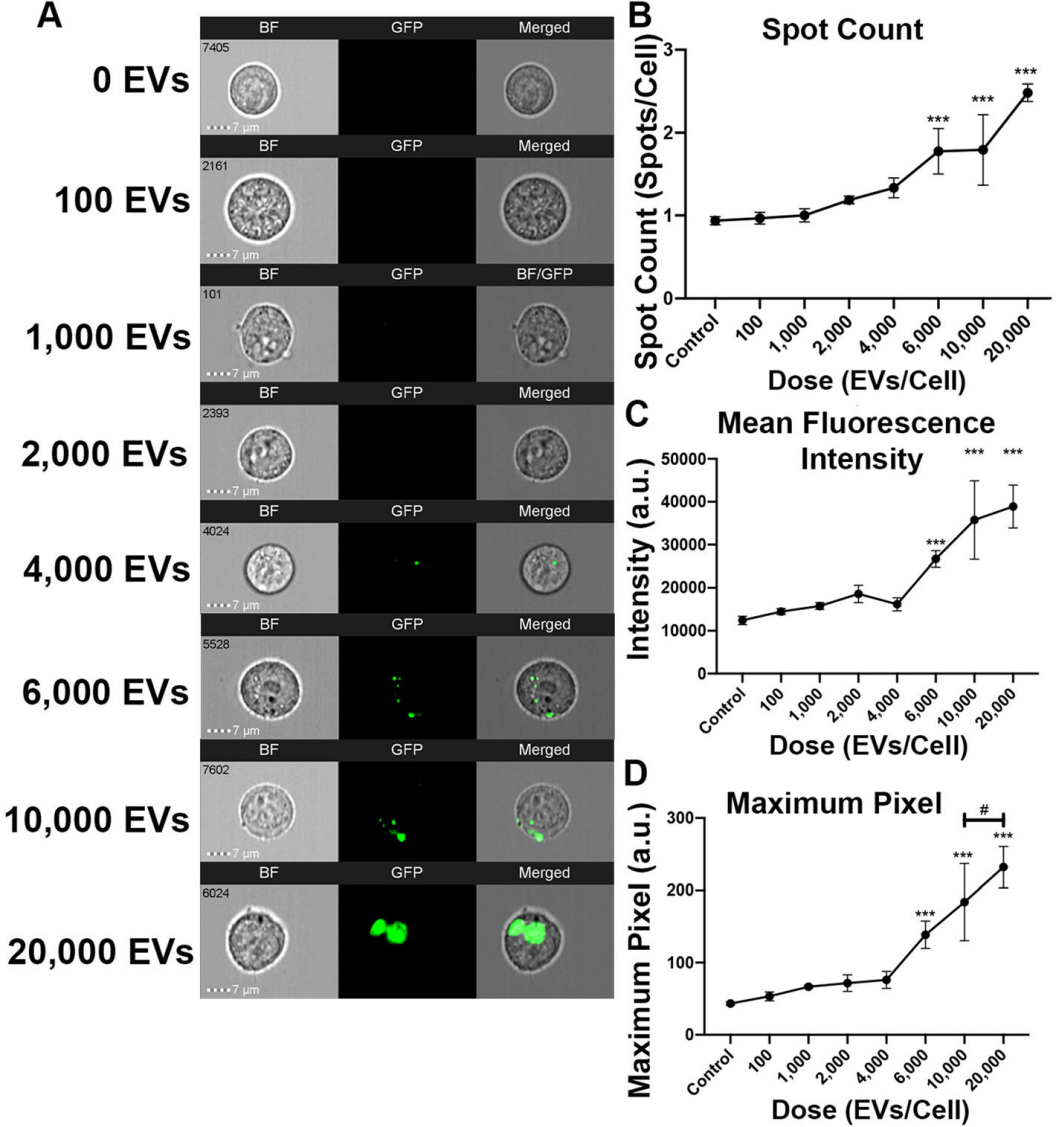
HEK293T EV uptake has a dose effect with a minimum threshold of 6000 EVs. HEK293T cells were co-cultured with HEK293T EVS at increasing doses from 0 to 20,000/cell. **a** Representative IFC images of recipient cells with respective EV doses. GFP localization signifies HEK293T EV uptake. **b**–**d** Quantification of dose assays compared with controls and each group via spot count, mean fluorescence intensity, and maximum pixel ratios. Bars represent mean ± SEM; *N* = 3; one-way ANOVA followed with Tukey’s post hoc test. *p < 0.05; ***p < 0.01

To develop a standard dose curve for the IFC platform, HEK293T EVs were co-cultured with HEK293T recipient cells at increasing doses ranging from 0 to 20,000 EVs per cell at 37 °C. Representative IFC images exhibited a visual increase of eGFP fluorescence with elevated doses of EVs (Fig. 3a). The lowest number of EVs that could be detected was 6000 EVs per co-cultured HEK293T cell. At this level, spot count (*p* = 0.0012), intensity (*p* = 0.0075), and maximum pixel (*p* = 0.0005) measurements were significantly greater than recipient cells without EVs (Fig. 3b–d). Therefore, doses of 6000 HEK293T EVs is the low threshold for uptake in our experimental condition. Similarly, doses of 10,000 and 20,000 EVs had higher spot count (10,000: *p* = 0.0009; 20,000: *p* < 0.0001), intensity (10,000: *p* < 0.0001; 20,000: *p* < 0.0001) and maximum pixel (10,000: *p* < 0.0001; 20,000: *p* < 0.0001) compared with cells without EVs. Comparing between the higher doses, there are no significant differences in spot count (6000 vs. 10,000: *p* = 0.999, 10,000 vs. 20,000: *p* = 0.0927), intensity (6000 vs. 10,000: *p* = 0.8482, 10,000 vs. 20,000: *p* = 0.999), and maximum pixel count (6000 vs. 10,000: *p* = 0.6056, 10,000 vs. 20,000: *p* = 0.5281) between 6000 and 10,000, along with 10,000 vs. 20,000. Similarly, comparing between 6000 and 20,000, there is no statistical difference in spot count (*p* = 0.0787) and intensity (*p* = 0.8083). There is a significant difference in maximum pixel between 6000 and 20,000 (*p* = 0.0140). Overall, the yield curve displays a significant dose dependence in all parameters (spot, intensity, max pixel, *p* < 0.0001). These results indicate that HEK293T EV uptake is dose dependent with a minimum threshold of 6000 HEK293T EVs per cell.

### HEK293T EV Temporal Uptake

**Fig. 4:**
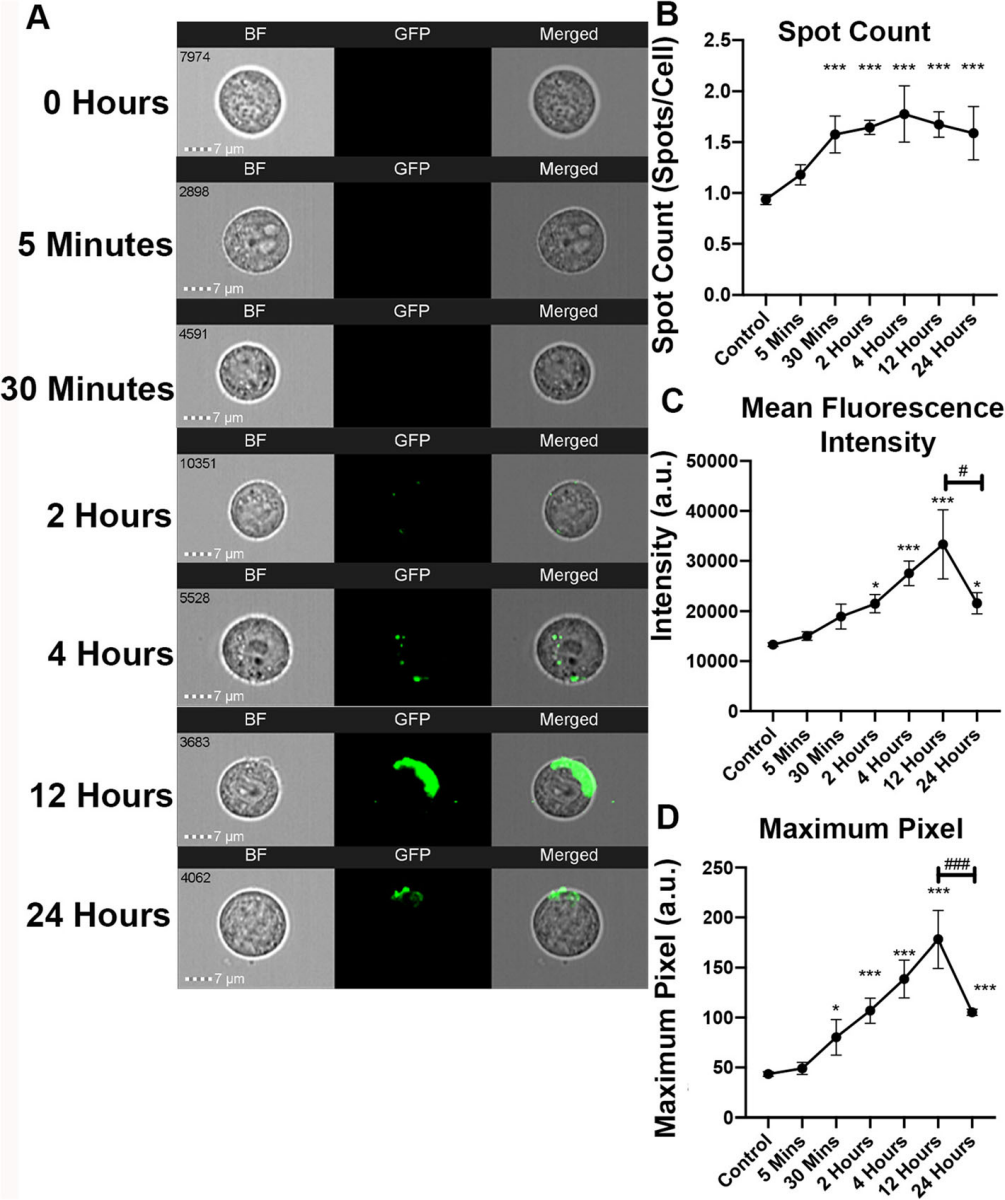
HEK293T EV uptake is time dependent. HEK293T cells were co-cultured with 6000 HEK293T EVs/cell for increasing lengths of time. **a** Representative IFC images of recipient cells, respectively. GFP localization signifies increased HEK293T EV uptake. **b**–**d** Quantification of time course assays compared with controls and each group via spot count, mean fluorescence intensity, and maximum pixel ratios. Bars represent mean ± SEM; *N* = 3; one-way ANOVA followed with Tukey’s post hoc test. *p < 0.05; ***p < 0.01

Using 6000 EVs per cell, HEK293T EVs were co-cultured with HEK293T cells for increasing lengths of time prior to IFC, ranging from 5 min to 24 h. Length of EV exposure played a key role in the amount of visible fluorescence in the recipient cells, declining after 12 h (Fig. 4a). Initially, 30 min of co-culture displayed a significant increase in spot count (*p* = 0.0081) suggesting a possible trend towards EV uptake, but not in other uptake parameters (intensity: *p* = 0.3073, max pixel: *p* = 0.0952) (Fig. 4b–d). At 2 h of co-culture, significantly higher spot count (*p* = 0.0028), intensity (*p* = 0.0420), and maximum pixel (*p* = 0.0006) were recorded compared with the recipient cells without EVs. Again, at 4 h of co-culture, all parameters were greater than controls (spot count: *p* = 0.0003, intensity: *p* < 0.0001, max pixel: *p* < 0.0001). Intensity and maximum pixel continued to be higher than controls at 4, 12, and 24 h of co-culture. There were no differences in any uptake parameters between 4 and 12 h of co-culture (spot: *p* = 0.999, intensity: *p* = 0.5797; maximum pixel: *p* = 0.2489). However, intensity (*p* = 0.0191), and maximum pixel (*p* = 0. 0027) decreased between 12 and 24 h of co-culture (Fig. 4 c,d ). Similar to the dose curve, HEK293T EV uptake is time dependent with consistent EV uptake at 4 h of incubation and a peak at 12 h. Collectively, a dose of 6000 EVs per cell seeded and a co-culture of 4 h has been standardized for the following uptake assays.

### Comparative Uptake of HEK293T EVs by Multiple Cell Lines

**Fig. 5:**
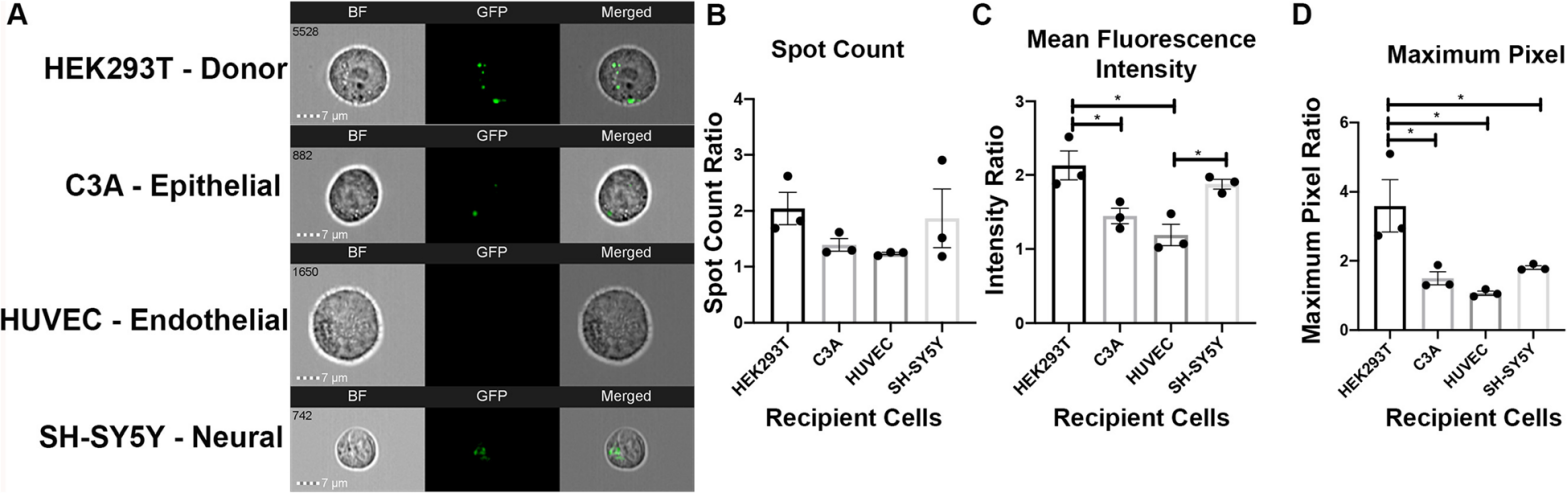
HEK293T EVs display uptake preference to HEK293T cells. HEK293T EVs were co-cultured with HEK293T cells, C3A epithelial cells, human umbilical vein endothelial cells (HUVEC), and SY5Y neural cells. **a** Representative IFC images of recipient cells co-cultured with HEK293T EVs. **b**–**d** Quantification of EV uptake preference assays compared with controls and each other via spot count, mean fluorescence intensity, and maximum pixel ratios. Bars represent mean ± SEM; *N* = 3; one-way ANOVA followed with Tukey’s post hoc test. *p < 0.05; ***p < 0.01

The hypothesis that EV uptake is a selective process where EVs are preferentially taken up by cells of their own origin was tested using IFC. HEK293T EVs were co-cultured with HEK293T cells or other cell lines: epithelial (C3A liver cells), endothelial (human umbilical vein endothelial cells), and neural (SH-SY5Y glioblastoma cells.). eGFP fluorescence is more abundant in HEK293T cells as compared with the other cell types (Fig. 5a). Compared with C3A and HUVECs, HEK293T cells had significantly higher fluorescence intensity (C3A: *p* = 0.0321; HUVEC: *p* = 0.0055) (Fig. 5c), when co-cultured with HEK293T EVs. Additionally, HEK293T cells had higher maximum pixel (C3A: *p* = 0.0221; HUVEC: *p* = 0.0079; SH-SY5Y: *p* = 0.0486) (Fig. 5d) as compared with all other recipient cell lines (Fig. 5b). Regarding intensity, SH-SY5Y cells were significantly higher than HUVECs when co-cultured with HEK293T EVs (*p* = 0.0304). These results support HEK293T EV selective uptake up by HEK293T cells compared with other cell lines in vitro.

### Differentiation Status of Neural Cells and HEK293T EV Internalization

**Fig. 6:**
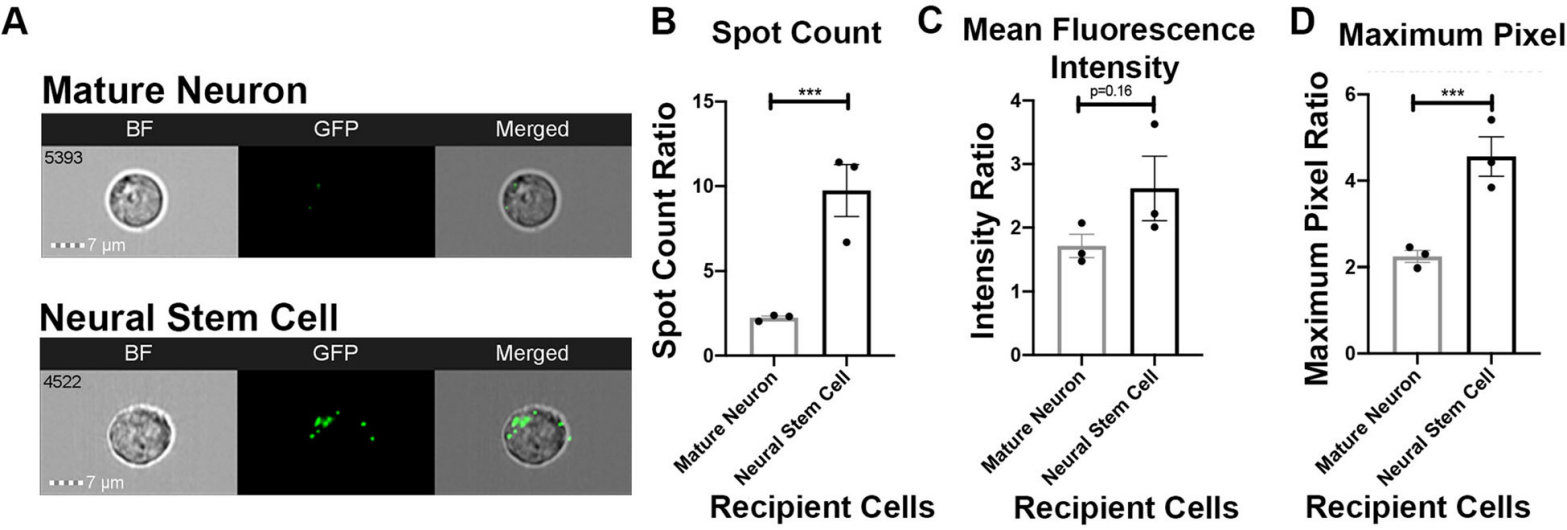
Neural differentiation status affects HEK293T EV uptake. HEK293T EVs were co-cultured with mature human neurons and human neural stem cells. **a** Representative IFC images of recipient cells co-cultured with HEK293T EVs. **b**–**d** Quantification of EV uptake preference assays compared with controls and each other via spot count, mean fluorescence intensity, and maximum pixel ratios. Bars represent mean ± SEM; *N* = 3; unpaired *T* test. *p < 0.05; ***p < 0.01 compared with 0 EVs (control)

Since EVs have been implicated for therapeutic and delivery purposes targeting neural diseases, human neural stem cells (hNSCs) and mature human neurons were used as recipient cell lines in our system to examine if the differentiation status of the recipient cell plays a role in selective uptake of EVs. Representative images from IFC displayed visual evidence of uptake in both cell types, but with the greatest eGFP localization in hNSCs (Fig. 6a). hNSCs co-cultured with HEK293T EVs have higher spot count (*p* = 0.0082) and max pixel (*p* = 0.0083) as compared to mature neurons. Together, these results suggest that differentiation status of neural cells affects uptake of HEK293T EVs.

## Discussion

### EV in vitro Uptake Standardization Process

A group of international experts on EVs emphasized a need to effectively determine the minimal effective dose of EVs for uptake assays, and here we have developed a system that can be effectively adopted by the field [26]. There are challenges when analyzing EV uptake. For example, as we and others observed, results can differ if the EV dose and exposure time are altered [26]. We addressed HEK293T EV dose and concentration as a kinetic variable. Also, an in vitro minimum effective dose may more uniformly predict in vivo biodistribution of EVs and be used to develop more consistent in vivo dosing parameters for EV therapeutics and delivery. In an in vivo mouse EV biodistribution study, increasing dose of HEK293T EVs resulted in a shift of the relative EV distribution in organs [27]. Similar to findings in a prior in vitro study using bladder cancer EVs [39], HEK293T EVs displayed a strong dose dependence with a minimal effective dose at 6000 EVs in our study. We are the first to use particles per cell as a sensitive dose measurement in vitro, which better correlates with in vivo models using particles per body weight. Our data also indicated a dose saturation limit after 6000 EVs, potentially informing future in vivo dose-ranging studies by indicating that higher doses may have limited benefits.

Another confounding variable of measuring EV uptake is the potential temporal effects on EV uptake. In our system, we found strong time dependence with uptake as early as 2 h with a potential decrease between 12 and 24 h. Similar to our findings, time dependence was reported in few studies using bladder cancer cells, tumor cells, and others with uptake as early as 15 min through 24 h [29–33, 39, 43, 49]. As seen with HEK293T EVs, the lower values at 24 h of co-culture may be a result of cell division or recycling/degradation of EVs internalized at early time points [50]. Specifically, since EVs have been shown to be internalized then broken down or internalized then released after 24 h, longer incubations may generate inaccurate internalization readouts [31, 50]. Our study is the first to use IFC to provide visual and quantitative evidence of a time-dependent yield curve on HEK293T EV uptake.

As the ISEV position paper suggests, the choice of an EV label may affect uptake, necessitating less disruptive techniques such as the GFP tagging methods used in our study. Specifically, 72% of researchers participating in a survey claim that lipid dye experiments are unreliable unless proper controls are used [26]. EV dyes do not reliably correlate with small EV content and may even increase vesicle size. Contamination of mislabeled lipoproteins and protein content and dye aggregation contributed to false positives [51, 52]. Therefore, we fused CD63 with an eGFP to label the HEK293T EVs. Similar to other reports of protein tagging, HEK293T EVs were GFP positive with no observed differences in diameter and maintained standard EV surface protein composition [38, 46]. Despite this, it is important to note that labeling EVs with specific EV proteins may limit the tracking to only a few subtypes of EVs expressing the respective markers. Other potential limitations may be that the fluorescence intensity is dependent on protein expression level, the efficiency of EV membrane labeling, and excitation strength of the light source [53]. However, IFC is sensitive, detecting low fluorescence intensity with accurate visualization of CD63-GFP particles at the 100-nm range [38, 54].

### Selective Uptake

EVs display proteins and other signals that may confer selective uptake [22, 23, 55]. Since the first step of EV biogenesis is the invagination of the plasma membrane, the EV membrane contains similar proteins, receptors, adhesion molecules, and integrins when compared with the donor cell membrane [22, 24, 55]. The lipid composition and tetraspanin proteins on EV membranes regulated by donor cells may contribute to EV tropisms with recipient cells [23, 34, 56]. MSC EVs selectively transported contents into MSCs, despite closer proximity to monocytes [24]. In contrast, others report that natural EVs were taken up equally by any cell type, regardless of EV origin [11, 22, 25, 57] when utilizing imaging or functional knockdown assays. Using the IFC platform, we found that HEK293T extracellular vesicles are taken up at greater quantities by HEK293T cells than other reported cell lines, thus suggesting an inherent EV uptake specificity. Through this outcome and the versatility of IFC, EV sources can be appropriately selected and analyzed for targeting specific recipient cells. To our knowledge, this is the first study utilizing imaging flow cytometry to analyze the specificity of HEK293T EVs.

In addition to self-selectivity, differentiation status of recipient cells has been hypothesized to play a role in uptake of EVs [32, 58, 59]. As our group and others have shown, EVs have therapeutic effects in the central nervous system and are known to modulate cell functions in neuronal development and adults [3, 7–9, 60]. Here for the first time, differentiation status of neurons affected EV uptake, where human neural stem cells had significantly greater uptake of HEK293T EVs compared to mature neurons. Immature hNSCs more actively internalize exogenous EVs than quiescent mature neurons. Since hNSCs are highly proliferative cells in culture, they may nonspecifically internalize nutrients and EVs. Similarly, immature dendritic cells internalized EVs at higher levels than mature dendritic cells [32, 59]. However, another study with myeloid precursor cells found that the mature dendritic cells and macrophages internalized more EVs than immature dendritic cells and monocytes [58]. The observed differences can be attributed to the phagocytic activity of further differentiated myeloid cells. Due to the in vitro evidence supporting selective uptake, HEK293T EVs can be used to modulate undifferentiated neurons in future therapeutic applications.

## Conclusions

In summary, we have further developed a quantitative and high-throughput platform for quantifying HEK293T EV uptake kinetics. This platform can be extended to other donor EVs and recipient cell types and assays for liposomes and synthetic nanoparticle delivery vectors. Significantly, we found that HEK293T EV uptake is a selective process, with specificity towards HEK293T cells. The IFC assays developed here can be used to better define parameters used in in vivo dose escalation and biodistribution studies and provide instrumental information for a predictive model of EV uptake outcomes in vivo.

## Data Availability

The datasets generated and/or analyzed during the current study are available from the corresponding author on reasonable request.

## Abbreviations

EV: Extracellular vesicles
HEK293T: Human embryonic kidney cell line
IFC: Imaging flow cytometry
hNSC: Human neural stem cell
GFP: Green fluorescent protein
BF: Bright field
SSC: Side scatter
ISEV: International Society of Extracellular Vesicles
